# Partner of Sld five 3: a potential prognostic biomarker for colorectal cancer

**DOI:** 10.1186/s13000-014-0217-5

**Published:** 2014-11-18

**Authors:** Xiaoli Sun, Wu Sui, Miaoling Huang, Yeli Wang, Yuanjie Xuan, Zaiqiu Wang

**Affiliations:** Department of Laboratory, Yuhuangding Hospital, No. 20 Yuhuangding East Road, Yantai, 264000 Shandong China; General Surgery, Yuhuangding Hospital, No. 20 Yuhuangding East Road, Yantai, 264000 Shandong China; Anorectal Surgery, Yuhuangding Hospital, No. 20 Yuhuangding East Road, Yantai, 264000 Shandong China

**Keywords:** Partner of Sld five 3, Colorectal cancer, Overall survival

## Abstract

**Background:**

Partner of Sld five 3 (PSF3) is a member of the evolutionarily conserved heterotetrameric complex “Go-Ichi-Ni-San” (GINS), which consists of SLD5, PSF1, PSF2, and PSF3. Previous studies have suggested that some GINS complex members are upregulated in cancer, but the status of PSF3 expression in colorectal cancer has not been investigated.

**Methods:**

We investigated the status of PSF3 expression in 137 consecutive resected colorectal caners by quantitative reverse-transcription polymerase chain reaction. Univariable and multivariable Cox regression analyses were performed to assess independent prognostic factors for overall survival in colorectal cancer.

**Results:**

In 137 restected colorectal cancer samples, median messenger RNA (mRNA) expression levels of PSF3 were significantly higher in tumor tissues (1.35 × 10^−3^, range 2.88 × 10^−4^ to 3.16 × 10^−2^) than in adjacent normal tissues (2.94 × 10^−4^, range 5.48 × 10^−5^ to 1.27 × 10^−3^) (*P* < 0.05). Moreover, high expression of PSF3 in tumor tissues was associated with shorter disease-free survival and overall survival. When analyzed with a Cox regression model, the PSF3 expression was an independent prognostic factor for overall survival. In addition, in patients with early stage (stage I and II) colorectal cancer, the overall survival rate of the high PSF3 expression group was significantly lower than that of the low PSF3 expression group (*P* < 0.001).

**Conclusions:**

The PSF3 expression plays an important role in the progression of colorectal cancer and acts as a factor significantly affecting the prognosis of patients.

**Virtual Slides:**

The virtual slide(s) for this article can be found here: http://www.diagnosticpathology.diagnomx.eu/vs/13000_2014_217

## Background

Partner of Sld five 3 (PSF3) is a member of the highly evolutionarily conserved tetrameric complex termed Go-Ichi-Ni-San (GINS), composed of SLD5, PSF1, PSF2, and PSF3. In yeast, the GINS complex associates with the Minichromosome maintenance (MCM) 2–7 complex and CDC45, and this “CMG complex” (CDC45/MCM2-7/GINS) regulates both the initiation and progression of DNA replication [1-3]. Thus, it has been suggested that GINS is involved in DNA replication in Xenopus and human [4-6]. However, a recent study suggested that PSF1/PSF2 is associated with the response to replication stress and acquisition of DNA damage in untransformed human dermal fibroblasts [7]. As it has been reported that DNA replication-associated protein in yeast has diverse functions in different cells, e.g. origin recognition protein Orc1 has a role in determining centrosome copy number [8], the exact functions of GINS components in mammalian cells are not yet clear.

Several recent reports have suggested that PSF1 is required for the acute proliferation of cells, particularly immature cells such as stem cells and progenitor cells and that this protein is useful in the successful detection of cancer stem cells [9-12]. Moreover, previous studies have suggested that some GINS complex members are upregulated in cancer, and some GINS components may be useful in the detection of cancer stem cells [13,14].

Although several studies have suggested that GINS components play a role in cancer [15,16], the expression status of these components in patients with colorectal cancer has not yet been examined. Therefore, we sought to evaluate the mRNA expression status of PSF3 in surgically resected samples of colorectal cancer tissue. We also investigated whether PSF3 expression in tumor tissues influenced the prognosis of these patients.

## Methods

### Patients

The study population comprised 137 consecutive patients (79 males and 58 females) who were examined and treated at Yuhuangding Hospital between January 2008 and December 2012 for colorectal cancer. All cases underwent complete resection in this study. Details of the clinical and demographic information, prognostic factors, and disease progression were collected prospectively. Of the 137 patients, 46, 54, 22, and 15 had stage I, II, III, and IV tumors, respectively. Forty-two patients were administered postoperiative adjuvant chemotherapy every three weeks for six months (Oxaliplatin 130 mg/m^2^ d1 + Capecitabine 1000 mg/m^2^ d1-d14). The study protocol was approved by the institutional review board of Yuhuangding Hospital and the study was conducted according to the principles of the Declaration of Helsinki. All patients provided written informed consent.

### RNA isolation and qRT-PCR

Quantitative reverse-transcription polymerase chain reaction (qRT-PCR) was used to determine the PSF3 expression level. Briefly, total RNA was extracted with Trizol reagent (Invitrogen, Grand Island, NY, USA) and dissolved in water according to the manufacturer’s instructions. Relative complementary DNA (cDNA) quantitation for PSF3 and an internal reference gene (β-actin) was done using a fluorescence-based, real-time detection method. The sequences of the primer used were as follows: PSF3 forward 5′-TGACAGTCCCGAGAATGCAGA-3′ and reverse 5′-TGCCTACCAGGGCTGAAGTG-3′; β-actin (internal reference gene) forward 5′-TGGCACCCAGCACAATGAA-3′, reverse 5′-CTAAGTCATAGTCCGCCTAGAAGCA-3′. The PCR mixture consisted 1200 nmol/l primer, 200 nmol/l probe, 200 nmol/l each of deoxyadenosine triphosphate, deoxycytidine triphosphate, deoxyguanosine triphosphate, deoxythymidine triphosphate, 3.5 mmol/l MgCl_2_, and × 1 Taqman Universal PCR Master mix to a final volume of 20 μl (all reagents were from PE Applied Biosystems, Foster City, CA). Cycling conditions were 95°C for 35 s and 60°C for 30s, followed by 40 cycles at 95°C for 15 s and 60°C for 1 min. Relative gene expression levels are expressed as ratios (differences between the Ct values) between two absolute measurements (PSF3/β-actin).

### Follow-up

The follow-up period was defined as the interval between the date of operation and the date of the patient’s death or the last visit. The follow-up time ranged from 7 months to 73 months (median, 41 months). All patients were followed until May 2014 with a follow-up rate of 100%. Disease-free survival was measured from the day of surgery to the day of the first evidence of tumor recurrence or metastasis. Overall survival was measured from the day of surgery to the day of death.

### Statistical analysis

Associations between PSF3 expression in tumor tissue and clinicopathological features were determined using the χ^2^-test. Survival was examined using the Kaplan-Meier method, and the significance of the difference was evaluated by a log-rank test. A Cox regression analysis was carried out to assess independent prognostic factors for disease-free survival and overall survival in colorectal cancer. All statistical calculations were performed using SPSS software (SPSS 17.0, Chicago, IL, USA) and *P* <0.05 was considered statistically significant.

## Results

### PSF3 expression level in colorectal cancer

The mRNA expression level of PSF3 were determined in 137 colorectal cancer and the adjacent normal tissues by qRT-PCR. Median mRNA expression levels were 1.35 × 10^−3^ (range 2.88 × 10^−4^ to 3.16 × 10^−2^) for tumor tissues and 2.94 × 10^−4^ (range 5.48 × 10^−5^ to 1.27 × 10^−3^) for adjacent normal tissues, and the differences were statistically significant (*P* < 0.05). To evaluate the role of PSF3 in colorectal cancer, we investigated whether PSF3 expression was associated with any of clinicopathological variables in the 137 enrolled cases of colorectal cancer. By adopting cut-off value according to median PSF3 expression level, we found that PSF3 expression was significantly associated with tumor size, depth of wall invasion, lymph node metastasis, TNM stage, tumor differentiation, and five-year survival. No significant relationship was noted between PSF3 expression and age, gender, distant metastasis, and adjuvant chemotherapy (Table 1).

**Table 1:** **Association between mRNA expression of PSF3 and clinicopathological characteristics in 137 patients with colorectal cancer**

**Variables**	**Total**	**PSF3 expression**		***P*** **-value**
		**Low level**	**High level**	
No. of patients	137	66	71	
Age in years, mean ± SD (range)	61.2 ± 7.6 (37–86)	59.4 ± 7.1 (37–81)	63.5 ± 8.8 (42–86)	0.634
Gender, M/F	89/48	42/24	47/24	0.728
Tumor size				0.013*
<5 cm	105	55	50	
≥5 cm	32	11	21	
Depth of wall invasion				0.006*
Tis-T2	81	43	38	
T3-T4	56	23	33	
Lymph node metastasis				0.047*
Negative	76	39	37	
Positive	61	27	34	
Distant metastasis				0.125
No	118	58	60	
Yes	19	8	11	
TNM stage				0.008*
I/II/III/IV	46/54/22/15	28/26/8/4	18/28/14/11	
Differentiation				0.021*
Well/Moderate	94	52	42	
Poor	43	14	29	
Adjuvant chemotherapy				0.079
Yes	42	22	20	
No	95	44	51	
Five-year survival				0.002*
Yes	58	35	23	
No	79	31	48	

*M/F* Male/Female, *PSF3* partner of Sld five 3, *SD* standard deviation.

*Significant *P*-value.

### High expression of PSF3 was associated with poor patient prognosis

**Figure 1:**
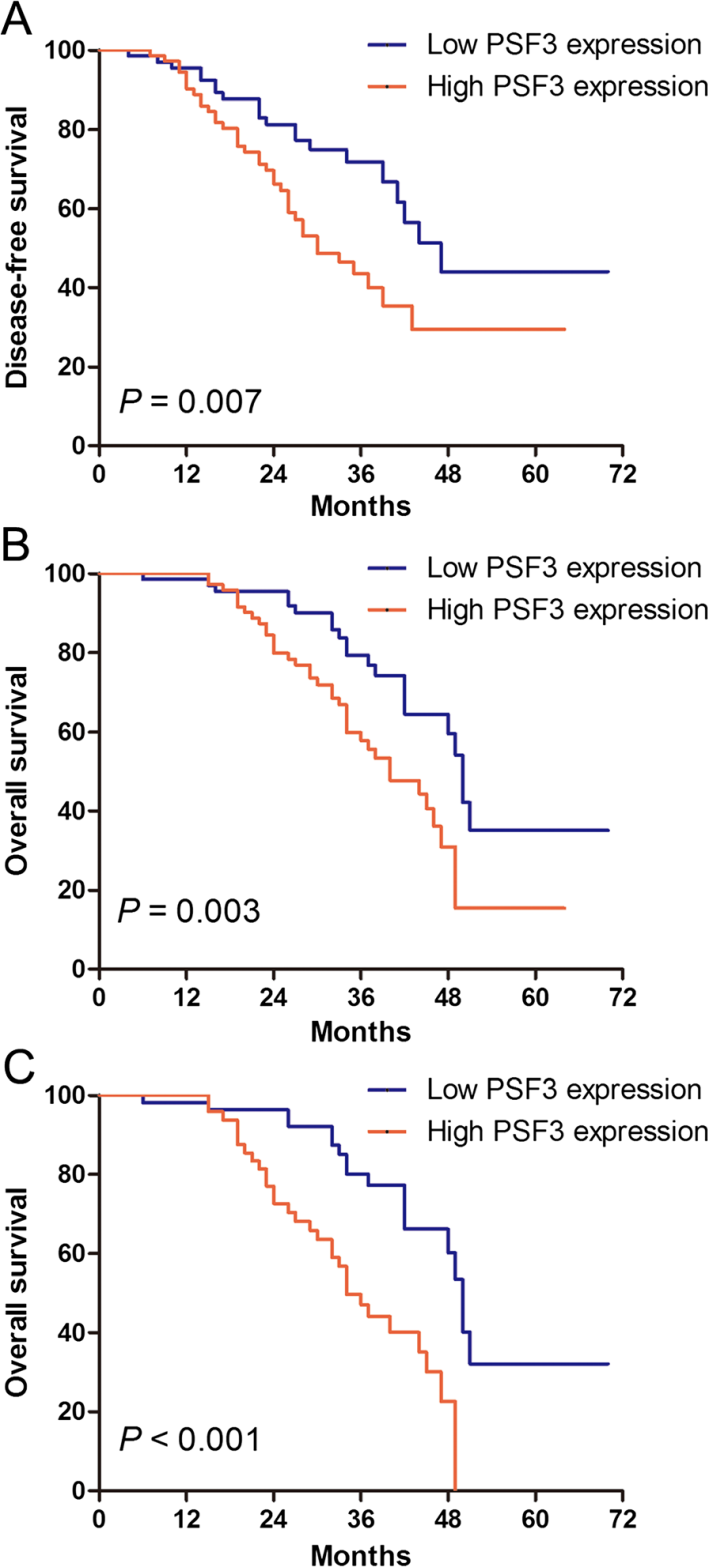
**Kaplan-Meier estimates of disease-free survival (A) and overall survival (B) for all patients and overall survival for early stage colorectal cancer patients (C).**

Using the data collected from 137 patients, we evaluated their prognosis and its relationship to the expression of PSF3. The disease-free survival in patients with low PSF3 levels (39.5 ± 7.2 months) was significantly longer than that in patients with high levels (28.6 ± 6.4 months) (*P* = 0.007; Figure 1A). Univariate analysis initially included age, gender, tumor size, depth of wall invasion, lymph node metastasis, distant metastasis, tumor differentiation, TNM stage, adjuvant chemotherapy, and PSF3 expression level for disease-free survival analysis. The tumor size, lymph node metastasis, distant metastasis, TNM stage, tumor differentiation, adjuvant chemotherapy, and PSF3 expression level were associated with disease-free survival and were introduced into the multivariate analysis (Table 2). In the multivariate analysis, late TNM stage, poor differentiation, and high PSF3 level were shown to have a statistically independent prognostic value with respect to disease-free survival (Table 2). In addition, we also examined the overall survival of PSF3 low level and PSF3 high level groups and found a statistically difference between the two groups by using the log-rank test (*P =* 0.003). The median survival time of patients with low PSF3 levels (59.7 ± 13.8 months) was significantly longer than that of patients with high PSF3 levels (47.2 ± 11.4 months) (Figure 1B). A univariate analysis indicated that among the clinicopathological factors, tumor size, lymph node metastasis, distant metastasis, tumor differentiation, TNM stage, adjuvant chemotherapy, and PSF3 expression level were correlated with the outcome (Table 3). Further assessment using the Cox multivariate analysis indicated that distant metastasis, poor differentiation, late TNM stage, and high PSF3 expression were statistically significant predictors for poor overall survival (Table 3).

**Table 2:** **Univariate and multivariate analyses of the association between the disease-free survival of 137 patients with colorectal cancer**

**Variables**	**Univariate analysis**		**Multivariate analysis**	
	**HR (95% CI)**	***P*** **-value**	**HR (95% CI)**	***P*** **-value**
Age	1.02 (0.94-1.13)	0.748		
Gender	0.95 (0.87-1.09)	0.396		
Tumor size	1.32 (1.03-1.58)	0.044	1.25 (0.98-1.46)	0.067
Depth of wall invasion	1.14 (0.96-1.35)	0.185		
Lymph node metastasis	1.74 (1.23-2.28)	0.006	1.27 (0.94-1.54)	0.083
Distant metastasis	1.56 (1.19-1.77)	0.013	1.33 (0.96-1.67)	0.075
TNM stage	1.91 (1.54-2.60)	<0.001	1.78 (1.21-2.13)	0.008*
Differentiation	1.66 (1.31-2.05)	0.002	1.45 (1.10-1.78)	0.028*
Adjuvant chemotherapy	1.25 (1.06-1.47)	0.037	1.08 (0.87-1.29)	0.154
PSF3 expression	1.83 (1.55-2.17)	<0.001	1.66 (1.36-2.02)	0.003*

*CI* confidence interval, *HR* hazard ratio, *PSF3* partner of Sld five 3.

*Significant *P*-value in multivariate analysis.

**Table 3:** **Univariate and multivariate analyses of the association between the overall survival of 137 patients with colorectal cancer**

**Variables**	**Univariate analysis**		**Multivariate analysis**	
	**HR (95% CI)**	***P*** **-value**	**HR (95% CI)**	***P*** **-value**
Age	1.12 (0.94-1.31)	0.576		
Gender	0.99 (0.87-1.14)	0.834		
Tumor size	1.41 (1.13-1.68)	0.033	1.04 (0.89-1.18)	0.473
Depth of wall invasion	1.33 (0.98-1.70)	0.065		
Lymph node metastasis	1.47 (1.08-1.85)	0.014	1.24 (0.96-1.47)	0.085
Distant metastasis	1.54 (1.22-1.92)	0.007	1.38 (1.12-1.63)	0.006*
TNM stage	1.67 (1.39-1.94)	<0.001	1.48 (1.15-1.77)	0.004*
Differentiation	1.41 (1.18-1.65)	0.009	1.26 (1.02-1.51)	0.041*
Adjuvant chemotherapy	1.37 (1.05-1.74)	0.023	1.14 (0.91-1.39)	0.093
PSF3 expression	1.59 (1.26-1.95)	<0.001	1.35 (1.10-1.64)	0.002*

*CI* confidence interval, *HR* hazard ratio, *PSF3* partner of Sld five 3.

*Significant *P*-value in multivariate analysis.

### High expression of PSF3 was also associated with poor patient prognosis in early stage colorectal cancer

In the current study, we further analyzed the association of PSF3 expression in early stage (stage I and II) colorectal cancer. Among the early stage cases, 46 and 54 patients were classified as high PSF3 level and low PSF3 level, respectively. A survival analysis that included only early stage patients revealed that the overall survival for the low PSF3 expression group was longer than that for the high PSF3 expression group. The log-rank test showed that the intergroup difference was statistically significant (*P* < 0.001; Figure 1C).

## Discussion

PSF3 is a member of the GINS complex, along with Sld5, PSF1, and PSF2. PSF1 is tightly regulated at the transcriptional level in stem cells and enables the successful detection of cancer stem cells [9-12]. Therefore, it seems reasonable that other GINS components may also facilitate the detection of cancer stem cells in tumors. Cancer stem cells, which are resistant to anti-cancer drugs and irradiation, appear to be responsible for tumor growth in hematological and solid cancers. The detection of these cells is critical for identifying molecular targets to inhibit their growth. Previous study has shown that all GINS components are overexpressed in intrahepatic cholangiocarcinoma tissues and PSF3 is also increased in lung adenocarcinoma [17]. To our knowledge, this is the first study to detect PSF3 expression in colorectal cancer and to show that PSF3 expression might be a useful prognostic marker for assessing patient survival in colorectal cancer.

In this study, we performed qRT-PCR of surgically resected colorectal cancer specimens to determine the PSF3 status in cancer tissues clinically. The results revealed that PSF3 expression was higher in the colorectal cancer specimens than in adjacent normal tissues. In order to elucidate the role of high PSF3 expression on the prognosis of patients with colorectal cancer, a prognostic analysis was carried using the patients’ follow-up data. Survival analysis revealed that the disease-free survival and overall survival in patients with low PSF3 expression was notably longer than that of patients with high PSF3 expression. These findings indicated that high PSF3 expression significantly affected the clinical course and was correlated with malignant behavior of tumors. The significance of PSF3 expression on these clinical features was also supported by our analysis of the relationship between PSF3 expression and clinicopathological characteristics of 137 patients. Cox multivariate analysis indicated that high PSF3 expression was the most significant predictor of poor prognosis, rather than the TNM stage or tumor differentiation. Furthermore, a prognostic analysis that included only early stage cases (stage I and II) revealed that the overall survival rate of the high PSF3 expression group was significantly lower than that of the low PSF3 expression group. These findings suggest that high PSF3 expression may be used as a reference index for molecular staging of patients with a high risk of death and thereby likely to benefit from intensive adjuvant therapy.

What is the basis of the relationship between high PSF3 expression and poor prognosis? We believe that high PSF3 expression may be related to cancer cell proliferation because PSF3 was required in the early stage of DNA replication, along with other GINS members [9-12]. A previous study by Nagahama et al. found high expression of PSF3 in several colon carcinoma cell lines (HCT116, colo320DM, SW837, and HT-29) and that PSF3 gene knock-down in these cell lines resulted in growth inhibition characterized by delayed S-phase progression [16]. The results suggested that PSF3 marks malignant colon cancer and has a role in cancer cell proliferation.

## Conclusions

In conclusion, we have shown that high PSF3 expression plays an important role in the progression of colorectal cancer and acts as a factor significantly affecting the prognosis of patients. These results suggested that PSF3 could be used as a reference index for the molecular staging to select patients at high risk of death and relapsed patients who may benefit from intensive adjuvant therapy.

## Abbreviations

PSF3: Partner of Sld five 3
GINS: Go-Ichi-Ni-San
mRNA: Messenger RNA
MCM: Minichromosome maintenance
qRT-PCR: Quantitative reverse-transcription polymerase chain reaction
cDNA: Complementary DNA

## References

[1] Takayama Y, Kamimura Y, Okawa M, Muramatsu S, Sugino A, Araki H (2003). GINS, a novel multiprotein complex required for chromosomal DNA replication in budding yeast. Genes Dev.

[2] Moyer SE, Lewis PW, Botchan MR (2006). Isolation of the Cdc45/Mcm2-7/GINS (CMG) complex, a candidate for the eukaryotic DNA replication fork helicase. Proc Natl Acad Sci U S A.

[3] Pacek M, Tutter AV, Kubota Y, Takisawa H, Walter JC (2006). Localization of MCM2-7, Cdc45, and GINS to the site of DNA unwinding during eukaryotic DNA replication. Mol Cell.

[4] Chang YP, Wang G, Bermudez V, Hurwitz J, Chen XS (2007). Crystal structure of the GINS complex and functional insights into its role in DNA replication. Proc Natl Acad Sci U S A.

[5] Kamada K, Kubota Y, Arata T, Shindo Y, Hanaoka F (2007). Structure of the human GINS complex and its assembly and functional interface in replication initiation. Nat Struct Mol Biol.

[6] Kubota Y, Takase Y, Komori Y, Hashimoto Y, Arata T, Kamimura Y, Araki H, Takisawa H (2003). A novel ring-like complex of Xenopus proteins essential for the initiation of DNA replication. Genes Dev.

[7] Barkley LR, Song IY, Zou Y, Vaziri C (2009). Reduced expression of GINS complex members induces hallmarks of pre-malignancy in primary untransformed human cells. Cell Cycle.

[8] Hemerly AS, Prasanth SG, Siddiqui K, Stillman B (2009). Orc1 controls centriole and centrosome copy number in human cells. Science.

[9] Ueno M, Itoh M, Kong L, Sugihara K, Asano M, Takakura N (2005). PSF1 is essential for early embryogenesis in mice. Mol Cell Biol.

[10] Ueno M, Itoh M, Sugihara K, Asano M, Takakura N (2009). Both alleles of PSF1 are required for maintenance of pool size of immature hematopoietic cells and acute bone marrow regeneration. Blood.

[11] Han Y, Ueno M, Nagahama Y, Takakura N (2009). Identification and characterization of stem cell-specific transcription of PSF1 in spermatogenesis. Biochem Biophys Res Commun.

[12] Nagahama Y, Ueno M, Miyamoto S, Morii E, Minami T, Mochizuki N, Saya H, Takakura N (2010). PSF1, a DNA replication factor expressed widely in stem and progenitor cells, drives tumorigenic and metastatic properties. Cancer Res.

[13] Obama K, Ura K, Satoh S, Nakamura Y, Furukawa Y (2005). Up-regulation of PSF2, a member of the GINS multiprotein complex, in intrahepatic cholangiocarcinoma. Oncol Rep.

[14] Nakahara I, Miyamoto M, Shibata T, Akashi-Tanaka S, Kinoshita T, Mogushi K, Oda K, Ueno M, Takakura N, Mizushima H, Tanaka H, Ohta T (2010). Up-regulation of PSF1 promotes the growth of breast cancer cells. Genes Cells.

[15] Ryu B, Kim DS, Deluca AM, Alani RM (2007). Comprehensive expression profiling of tumor cell lines identifies molecular signatures of melanoma progression. PLoS One.

[16] Nagahama Y, Ueno M, Haraguchi N, Mori M, Takakura N (2010). PSF3 marks malignant colon cancer and has a role in cancer cell proliferation. Biochem Biophys Res Commun.

[17] Hokka D, Maniwa Y, Tane S, Nishio W, Yoshimura M, Okita Y, Ohbayashi C, Sakai Y, Chen X, Hayashi Y (2013). Psf3 is a prognostic biomarker in lung adenocarcinoma. Lung Cancer.

